# Destabilizing COXIV in Müller Glia Increases Retinal Glycolysis and Alters Scotopic Electroretinogram

**DOI:** 10.3390/cells11233756

**Published:** 2022-11-24

**Authors:** Nana Yaa Nsiah, Denise M. Inman

**Affiliations:** Department of Pharmaceutical Sciences, North Texas Eye Research Institute, University of North Texas Health Science Center, Fort Worth, TX 76107, USA

**Keywords:** Müller glia, glaucoma, oxidative phosphorylation, metabolism, electroretinogram, glycolysis

## Abstract

Müller glia (MG), the principal glial cell of the retina, have a metabolism that defies categorization into glycolytic versus oxidative. We showed that MG mount a strong hypoxia response to ocular hypertension, raising the question of their relative reliance on mitochondria for function. To explore the role of oxidative phosphorylation (OXPHOS) in MG energy production in vivo, we generated and characterized adult mice in which MG have impaired cytochrome c oxidase (COXIV) activity through knockout of the COXIV constituent COX10. Histochemistry and protein analysis showed that COXIV protein levels were significantly lower in knockout mouse retina compared to control. Loss of COXIV activity in MG did not induce structural abnormalities, though oxidative stress was increased. Electroretinography assessment showed that knocking out COX10 significantly impaired scotopic a- and b-wave responses. Inhibiting mitochondrial respiration in MG also altered the retinal glycolytic profile. However, blocking OXPHOS in MG did not significantly exacerbate retinal ganglion cell (RGC) loss or photopic negative response after ocular hypertension (OHT). These results suggest that MG were able to compensate for reduced COXIV stability by maintaining fundamental processes, but changes in retinal physiology and metabolism-associated proteins indicate subtle changes in MG function.

## 1. Introduction

The retina has the highest per capita energy consumption of any tissue in the body [1]. Of note, metabolic dysfunction and energy deficits have been linked to neurodegeneration in many retinal diseases [2,3,4,5,6]. While most of the energy in the retina is used by neurons for electrical signaling and vision processing, glial cells are believed to support neuronal energy generation through the provision of metabolic intermediates such as lactate [7,8]. This relationship was proposed by the astrocyte–neuron lactate shuttle (ANLS) hypothesis which states that within the central nervous system (CNS), glial cells are primarily glycolytic, whereas neurons rely on the more efficient oxidative phosphorylation (OXPHOS) for energy generation [9]. Although evidence supports glial cells deriving energy from glycolysis in the CNS [10,11], energy generation in Müller glia (MG), the primary glial cells in the retina, is unique. Evidence for MG engaging in incomplete glycolysis [12] or the consumption of photoreceptor-derived lactate for OXPHOS [13] contradicts reports of MG being glycolytic cells [14,15,16,17]. Gaining insight into how MG balance and shift their metabolism in disease contexts such as glaucoma will be valuable for developing methods to address energy-deficit-associated neurodegeneration.

MG modulate retinal homeostasis and provide structural support [18]. By means of their unique morphology, they make close contact with all retinal neurons and mediate materials exchange between the retina and external regions, such as the vitreous and blood [19]. They regulate extracellular potassium ion (K^+^) and water balance, recycle glutamate and provide antioxidant defense via the synthesis and release of glutathione [20,21,22,23]. MG dysfunction has been implicated in neurodegeneration in various retinal diseases [23]. Injury to the retina is accompanied by reactive MG gliosis [21]. MG hypertrophy and altered protein expression during gliosis are early markers of retinal injury [22]. Reactive MG have been shown to be both protective and toxic to neurons [24]. Protective functions include enhanced expression of neuroprotective factors, such as pigment epithelium-derived factor (PEDF), a growth factor required for retinal ganglion cell survival, and glutathione, which enables neurons to manage oxidative stress [25,26]. On the other hand, detrimental effects involve decreased glutamate uptake and diminished extracellular potassium ion clearance [27]. The down-regulation of inward rectifying (K_ir_) channels impairs glutamate uptake and extracellular K^+^ removal by MG, causing neuronal hyper-excitability and retinal edema [28,29,30].

We recently observed that MG mount a strong hypoxia response to ocular hypertension [31,32]. This sensitivity to low levels of oxygen in the retina led us to examine what is known about mitochondria and OXPHOS in MG. Though the roles of MG in the retina have been studied extensively [19], there is some disagreement about how MG generate cellular energy. Even though MG are more resistant to cellular stressors than neurons, the inhibition of energy metabolism pathways has been shown to exacerbate neuronal damage under pathologic conditions [33,34]. Evidence for MG support of neuronal metabolism makes it imperative to understand their energy-generation pathways and how disease-associated changes may affect their function.

Early studies of MG bioenergetics supported the ANLS hypothesis. Poitry-Yamate et al. (1995) found higher levels of lactate in pure primary MG culture medium compared to MG and photoreceptor co-culture medium [14]. Further evidence came from in vitro studies of human MG, where inhibiting glycolysis caused the cells to die, while blocking OXPHOS did not affect cell survival or ATP levels [15].

More recent histological evidence suggests that MG may not be capable of glycolysis or OXPHOS. Using retinal cross-sections from adult mice, Lindsay et al. (2014) reported that MG were deficient in all isoforms of pyruvate kinase, the final rate-limiting enzyme for glycolysis. Additionally, assessing the rate of lactate production from whole retinas versus primary MG revealed that lactate release from MG was too slow to be the main source of lactate for neurons in the retina [34]. Other studies have shown that photoreceptors are the main sites of aerobic glycolysis in the retina [35], passing on lactate to MG for fuel [13]. MG have been shown to express low levels of hexokinase but high levels of tricarboxylic acid (TCA) cycle anaplerotic and cataplerotic enzymes [36]. Additionally, inhibiting glycolysis in MG in vivo did not affect their survival [12]. These data and our observations about MG sensitivity to hypoxia [31,32] suggest that MG may not be as reliant on glycolysis as previously reported.

To investigate the role of OXPHOS in MG in vivo, we crossed GLAST-Cre mice with Cox10-floxed mice to generate GLASTCreERT2:Cox10^fl/fl^ transgenic mice. Deleting Cox10, a farnesyltransferase required for cytochrome c oxidase (COXIV) assembly, impairs the electron transport chain and mitochondrial ATP synthesis [37,38]. We found that limiting OXPHOS in MG did not overtly affect their morphology, but there were alterations in retinal physiology and glycolytic profile. Interestingly, these MG changes were not sufficient to exacerbate ocular hypertension-associated loss of RGCs or photopic negative response.

## 2. Materials and Methods

### 2.1. Animals

All animal procedures were conducted in compliance the University of North Texas Health Science Center Institutional Animal Care and Use Committee (protocols 2019-0020 and 2022-0026) and conformed to the Association for Research in Vision and Ophthalmology (ARVO) statement for use of animals in ophthalmic and vision research. Mice used were the B6.Cg-Gt(ROSA)26Sor^tm14(CAG-tdTomato)Hze^/J (Jackson Labs Stock#007914), and the Tg(Slc1a3-cre/ERT)1Nat/J (Jackson Labs Stock #012586) strain crossed to the B6.129X1-Cox10^tm1Ctm^/J (Jackson Labs Stock #024697) to generate mice in which Cox10 was knocked out of every cell expressing Slc1a3 (GLAST, or glutamate aspartate transporter) upon induction of cre recombinase through tamoxifen injection (10 mg/mL in sunflower oil; IP). Knockout mice were homozygous for the Cox10^tm1Ctm^ allele and were carriers of the GLAST-cre/ERT. These mice are referred to as GLAST-COX10^fl/fl^. Age-matched GLAST-cre/ERT-COX10 wild-type (WT) and cre-negative homozygous Cox10 mice served as controls. We performed daily intraperitoneal injection of tamoxifen (100 mg/kg) for five consecutive days starting at P30 to induce transcription of cre-recombinase and subsequent elimination of exon 6 of the Cox10 gene. To detect the Cox10 floxed mice, ear or tail snips were sent to Transnetyx for genotyping using the following primers: Cox10 Forward Primer: GACCTGCAGCCCAAGCT, Reverse Primer: CGCCAGCATCTTCACATCCA. Cre was detected with Forward Primer: TTAATCCATATTGGCAGAACGAAAACG and Reverse Primer: CAGGCTAAGTGCCTTCTCTACA. Previous studies found that the half-life of mitochondria in adult rodent CNS is 24 –26 d (Beattie et al., 1967; Menzies and Gold, 1971), so we analyzed changes in the retina at a minimum of 2 months after cre induction, e.g., in 3-month-old mice.

### 2.2. PCR on Genomic DNA

Whole retina DNA was used to detect the recombined Cox10 allele following PCR with the primers Forward TGAGTAGAATGGCTTCCGGAAGGG and Reverse AGCAGCAAAGAGGGCTCACTTCTTGC. A 465 bp band confirms Cox10 genomic recombination.

### 2.3. Cell Culture

MG were isolated from 1-month-old mice after tamoxifen treatment. Dissected retinas were digested in papain with 200 U/mL of DNase (Worthington) and incubated at 37 °C for 10 min. Next, an equal volume of ovomucoid (Worthington) was added and retinas were gently triturated to obtain small pieces (approximately 1 mm × 1 mm). After centrifugation at 300× *g* for 10 min, tissue pieces were cultured in DMEM supplemented with 10% FBS (Sigma Aldrich, St. Louis, MI, USA) and 1% penicillin–streptomycin (Invitrogen, Carlsbad, CA, USA). The media was changed every 3 days. Cells were maintained in culture for 8 weeks before analysis.

### 2.4. Intraocular Pressure (IOP) Measurements

Ten IOP measurements per eye were taken in lightly anesthetized mice (2.5% isoflurane) using a TonoLab rebound tonometer (iCare Finland) prior to, and then weekly after, the induction of ocular hypertension (OHT).

### 2.5. Cryosectioning

Eyes were fixed with 4% paraformaldehyde (PFA) in phosphate-buffered saline (PBS). Adult mice were perfused with 4% PFA, and the eyes and brains were post-fixed for 1 h at room temperature. Tissue was immersed in 30% sucrose/PBS overnight and then embedded and frozen in OCT medium (Tissue-Tek). Using a Leica cryostat, fixed and cryoprotected retinas were sectioned sagittally at 8–10 μm, and stored at −70 °C until use.

### 2.6. Immunofluorescence (IF)

Cryosectioned retinas were immunolabeled using the antibodies as listed in Table 1. To determine the dilutions, optimization with different serial dilutions was performed. Retina sections were blocked in 5% donkey serum and 0.4% TritonX-100 in PBS for 1 h, then primary antibodies were incubated with the tissue overnight. After washing in 0.1M phosphate-buffered saline, and a further 30 min block, secondary antibodies (1:250; Alexa-Fluor 488, 594, or 647; Jackson ImmunoResearch, West Grove, PA, USA) prepared in block were added and incubated for 2 h. Sections were washed again in PBS, then cover-slipped using DAPI-Fluoromount-G (SouthernBiotech, Birmingham, AL, USA). We confirmed antibody specificity by incubating sections with secondary antibodies but without primary antibodies. RGC counts in the ganglion cell layer (GCL) were obtained from sagittal sections that included the optic nerve and optic nerve head using four sections per slide, and 5 slides per region using ImageJ. For 3NT and 8OHdG fluorescence density quantification, the freehand-selection tool was used to outline the inner nuclear layer (INL) to nerve fiber layer (NFL) area on each retinal section analyzed. The analyze tool was used to measure the mean pixel intensity. Mean fluorescence intensity values from the six wildtype and six mutant retinas were compared using unpaired student’s *t*-test.

### 2.7. Histochemistry

Sequential cytochrome c oxidase (COX) and succinate dehydrogenase (SDH) histochemistry was performed as described previously [39]. Briefly, 8 µm fresh–frozen retina cross sections were cut using a cryostat. Cryosections were incubated with COX medium containing reagents that were all obtained from Sigma-Aldrich (St. Louis, MO, USA): 100 µM cytochrome c, 1X diaminobenzidine tetrahydrochloride (DAB), and 20 µg/mL catalase at 37 °C for 40 min. Slides were washed 4 times, 10 min each time, in 0.1 M PBS pH = 7.0, followed by incubation with SDH medium containing 130 mM sodium succinate, 0.2 mM phenazine methosulphate, 1 mM sodium azide, and 1.5 mM nitroblue tetrazolium (NBT) in 0.1 M PBS, pH 7.0, for 30 min at 37 °C. For each genotype, three mice of either sex were analyzed.

### 2.8. Protein Analysis by Capillary-Based Electrophoresis

Cells and retina proteins were collected in RIPA buffer with 1% HALT protease and phosphatase inhibitors (Thermo Fisher Scientific, Waltham, MA, USA). The total protein concentration was measured using the Bicinchoninic Acid Assay Kit (Pierce, ThermoFisher Scientific) and read on a Cytation 5 (Biotek, Winooski, VT, USA) plate reader. Proteins were analyzed by capillary tube-based electrophoresis immunoassay using the ProteinSimple Jess instrument (Bio-Techne, Minneapolis, MN, USA) and each protein was normalized to total protein in the sample capillary. Antibodies used for protein analysis are listed in Table 1.

### 2.9. Electroretinography (ERG)

#### 2.9.1. Scotopic

ERG was recorded using the HMsERG system (Ocuscience Inc., Henderson, NV, USA). Mice were dark-adapted overnight and anesthetized with 2.5% isoflurane. Pupils were dilated with tropicamide and phenylephrine (Akron Pharmaceuticals, Lake Forest, IL, USA). A drop of 2.5% hydroxypropyl methylcellulose (Alcon Laboratories, Inc., Fort Worth, TX, USA) solution was placed on the cornea to keep it moistened and to provide an electrical contact with the ERG electrode. The mice were placed under the 76 mm diameter Ganzfeld dome to assure uniform illumination of the eyes. The eyes were exposed to a sequential increment of flash intensities (0.1 to 10 cd.s/m^2^) for 40 s with an interval between each flash. The data were analyzed using ERGVIEW (Ocuscience Inc., Henderson, NV, USA). The b-wave amplitude was measured from the trough of the a-wave to the peak of the b-wave.

#### 2.9.2. Photopic Negative Response

Electroretinography (ERG) was performed on both treated and untreated eyes at 5 weeks following microbead injection using an Espion Diagnosys system (Diagnosys LLC, Littleton, MA, USA). Tropicamide (0.5%, Akorn, Lake Forest, IL, USA) and 0.5% proparacaine hydrochloride (Falcon Pharmaceuticals, Fort Worth, TX, USA) were used for pupil dilation. Body temperature was maintained at 37 °C with a homeothermic controller and unit. Electrical signals were recorded with two 3 mm gold wire loop electrodes (Diagnosys) contacting the corneal surface of eyes precoated with a 2.5% hydroxypropyl-methylcellulose solution (Gonak, Akorn, Lake Forest, IL, USA). A subdermal needle electrode (Viasys Healthcare, Chicago, IL, USA) between the ears served as common reference while the other subdermal needle electrode inserted at the base of the tail acted as ground. Retinal responses were recorded simultaneously from both eyes. Light stimuli were delivered via a ColorDome unit. Three different light intensities, 1, 5 and 7 cd.s/m^2^, were used in a 3-step examination. The stimuli were blue flashes (6.28 cd/m^2^) and green (560 nm) background (10 cd/m^2^). Each recording was an average of 10 sweeps with an inter-stimulus interval of 0.4 s. Signals were band-pass filtered at 1 kHz. The PhNR amplitude was measured from the baseline to the trough following the b-wave.

### 2.10. Biochemical Assays

Retinas were isolated and flash frozen in liquid N2 or directly used for measurement of glycogen, glutathione, hexokinase or ATP. All the biochemical assays were performed according to the manufacturer’s instructions (see Table 2). Biochemical data were normalized by total protein level of each retina sample. Retina total protein was quantified using the Pierce™ BCA protein assay kit.

### 2.11. Ocular Hypertension (OHT) Model

Magnetic microbeads (1.5 μL, 8 μm diameter; COMPEL COOH-Modified, UMC4001; Bangs Laboratories, Inc., Fishers, IN, USA) were injected into the anterior chamber of both eyes via glass pulled micropipette attached to a microinjection system (World Precision Instruments, Sarasota, FL, USA), and a neodymium magnet was used to guide the microbeads into the trabecular meshwork to block the outflow of aqueous humor and increase IOP [32]. We used the magnetic bead model successfully to elevate IOP 4–5 weeks. To eliminate the confounding factor of contralateral eye effects on glial activation, separate animals served as control.

### 2.12. Statistics

Data were collected, and graphs made using GraphPad Prism v.9 (Dotmatics, Boston, MA, USA). Statistical analyses were performed after evaluating normality. When distributions were Gaussian, parametric unpaired two-tailed *t*-test was performed, with adjustments made when necessary for unequal variance by using Welch’s correction. Non-Gaussian distributions resulted in a non-parametric Mann–Whitney test to compare ranks. Electroretinography scotopic data were analyzed by two-way ANOVA with Sidak’s multiple comparisons test. *p* < 0.05 was considered statistically significant.

## 3. Results

### 3.1. Characterization of COX10 Knockout

**Figure 1:**
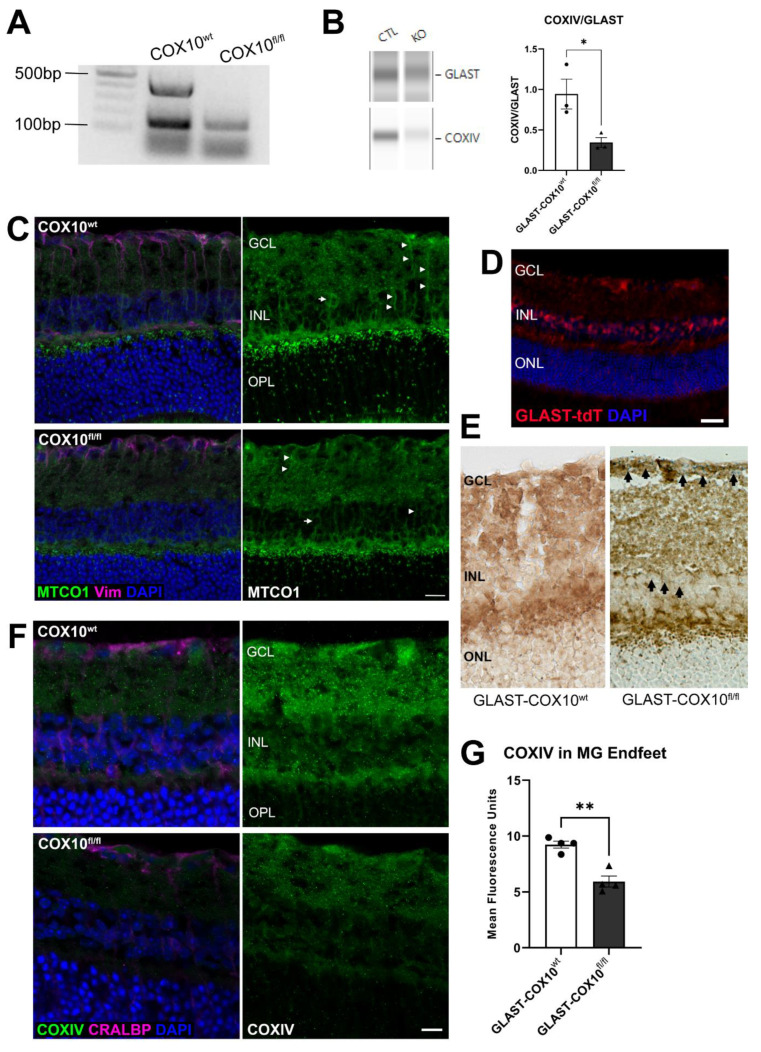
Recombination and Deletion of COX10. (**A**) Confirmation of Cox10 gene recombination in conditional knockout retinas at 3 months of age. An approximately 500 bp band indicates loss of exon 6 of the Cox10 gene. (**B**) Capillary electrophoresis lanes showing COXIV and GLAST protein levels in primary MG cell lysates from control and GLAST-COX10^fl/fl^ mice. Right: Quantitative analysis of protein levels shows a statistically significant decrease in COXIV in MG from GLAST-COX10^fl/fl^ mice (* *p* < 0.05). Error bars represent mean ± SEM for 3 biological replicates. (**C**) Immunofluorescence in retina cross-sections from 3-month-old wildtype control and GLAST-COX10^fl/fl^ mice immunolabeled with COX10 (green), CRALBP (magenta), and DAPI (blue). (**D**) Distribution of tdTomato labeling (red) as directed by the GLAST-cre locus, confirming the Müller glia-specific targeting of the COX10^fl/fl^ allele. (**E**) Representative histochemical images showing retina cross-sections from 3-month-old control and GLAST-COX10^fl/fl^ mice after COX/SDH histochemistry. The GLAST-COX10^fl/fl^ retina cross section (right) has blue chromophore product in the GCL (Müller glia endfeet) and INL (Müller glia cell bodies) indicating a loss of COX activity (black arrows). (**F**) Retina from wildtype and GLAST-COX10^fl/fl^ mice immunolabeled with COX10 (green), CRALBP (magenta), and DAPI. (**G**) Mean fluorescence of COXIV measured in MG endfeet using ImageJ showed a significant decrease in COXIV in the GLAST-COX10^fl/fl^ mice (** *p* = 0.0013). Error bars represent mean ± SEM. GCL = ganglion cell layer; INL = inner nuclear layer; ONL = outer nuclear layer. Scale bars: 25 µm.

To investigate the importance of oxidative phosphorylation to MG function in vivo, we crossed mice homozygous for floxed Cox10 with mice hemizygous for GLAST-cre. Following tamoxifen treatment of GLAST-COX10^fl/fl^ mice, we confirmed Cox10 gene recombination using PCR and genomic DNA from the retinas of 3-month-old mice. An approximately 500 bp band from the mutant retinas confirmed the loss of exon 6 from the Cox10 gene (Figure 1A). COX10 immunofluorescence indicated a significant loss of protein in the MG of the GLAST-COX10^fl/fl^ mice (Supplementary Data, Figure S1). COX10 protein is required for the assembly of cytochrome c oxidase (Complex IV, or COXIV), so we analyzed COXIV protein levels using capillary electrophoresis and immunohistochemistry. These analyses revealed that compared to control mice, primary MG cells from GLAST-COX10^fl/fl^ mice exhibited significantly lower levels of COXIV (Figure 1B; Supplementary Data, Figure S2). Protein levels for glutamate-aspartate transporter (GLAST), the promoter driving cre recombinase expression in these mice, were analyzed in Cox10^wt^ and Cox10^fl/fl^ retina; no difference was observed in GLAST protein across these genotypes (Supplementary Data, Figure S3). Cytochrome c oxidase subunit 1, (MTCO1) levels were lower in GLAST-COX10^fl/fl^ mouse retina cross sections compared to wildtype control GLAST-COX10^wt^ (Figure 1C). Figure 1D shows a retinal cross-section from a GLAST-cre mouse crossed to the Ai14 strain, resulting in tdTomato expression from the GLAST-cre locus. The tdTomato label indicates full targeting of MG by the GLAST-cre locus in the retina. To demonstrate the absence of COXIV activity in MG cells, we used sequential COX and succinate dehydrogenase histochemistry. Dark brown staining indicated normal COXIV function, while the appearance of blue-stained mitochondria marked COXIV deficient cells (Figure 1E) [39]. Blue puncta were observed in the GCL, corresponding to large concentrations of mitochondria in the Müller glia endfeet, and in the INL, where Müller glia cell bodies are observed (black arrows). Blue puncta could be observed in layers where Müller glia or their processes are found. COXIV protein was lower in the GLAST-COX10^fl/fl^ retina as compared to the control (Figure 1F). Quantification of mean fluorescence of COXIV in MG endfeet showed a significant decline in immunolabeling for COXIV in the GLAST-COX10^fl/fl^ mouse retina (Figure 1G). These various gene, protein, and histochemical measures indicated the successful destabilization of COXIV through the conditional knockout of Cox10 in GLAST-expressing cells, including MG.

### 3.2. Stress Effect of COX10 Knockout and ERG

**Figure 2:**
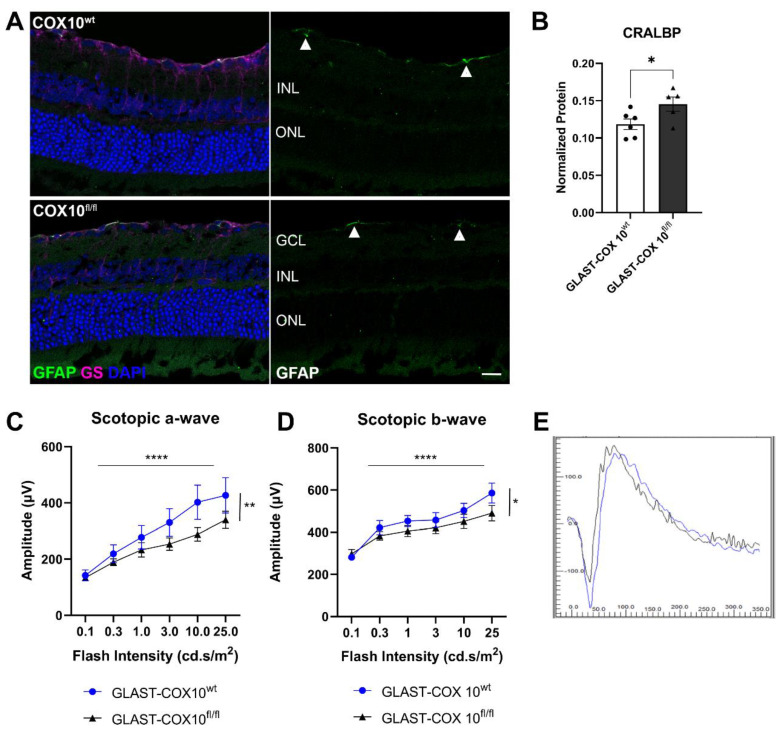
Selectively knocking down COX10 in MG did not induce gliosis but impacted scotopic ERG. (**A**) Immunofluorescence images showing GFAP (green) and glutamine synthetase (magenta) localization in retina cross-sections from 3-month-old mice from control and GLAST-COX10^fl/fl^ retinas. There was limited expression of GFAP in the nerve fiber layer, which likely corresponds to astrocytes (white arrowheads). GCL = ganglion cell layer; INL = inner nuclear layer; ONL = outer nuclear layer. (**B**) Capillary electrophoresis of wildtype and GLAST-COX10^fl/fl^ mouse retina shows an increase in the CRALBP protein in the conditional knockout mice (* *p* = 0.0459). Error bars represent mean ± SEM. (**C**) Knocking down COX10 in MG significantly reduced a-wave amplitude (two-way ANOVA; simple main effects for genotype (F(1,72) =7.946; ** *p* = 0.0062) and light intensity (F(5,72) =12.05; **** *p* < 0.0001)). Error bars represent mean ± SEM. (**D**) Knocking down COX10 in MG also significantly reduced the b wave amplitude. Two-way ANOVA to analyze the effect of genotype and light intensity on scotopic b-wave amplitude revealed that there were was not a statistically significant interaction between the effects of genotype and light intensity (F(5,72) = 0.67; *p* = 0.64). Simple main effects analysis showed that genotype (F(1,72) =5.65; * *p* = 0.02) and light intensity (F(5,72) =14.43; **** *p* < 0.0001) each had statistically significant effects on scotopic b-wave amplitude. Error bars represent mean ± SEM; N = 6/group. (**E**) Representative traces for scotopic ERG for wildtype and GLAST-COX10^fl/fl^ mice 8 weeks after tamoxifen injection. GCL = ganglion cell layer; INL = inner nuclear layer; ONL = outer nuclear layer. Scale bar: 25 µm.

As perturbing MG mitochondrial respiration through Cox10 knockout may induce retinal stress, we examined GFAP expression, an intermediate filament upregulated in MG during stress or injury. Representative immunofluorescence images are shown for 3-month-old control (COX10^wt^) and Cox10 knockout (COX10^fl/fl^) eyes (Figure 2A). There was no significant difference in GFAP expression between control and Cox10 knockout (KO) retinas. MG maintained their morphology as shown by glutamine synthetase (GS) immunolabeling (Figure 2A). The limited expression of GFAP was confined to the nerve fiber layer, which likely corresponds to astrocytes, suggesting that inhibiting mitochondrial respiration in MG did not induce reactive gliosis. Next, to determine the effect of MG COX10 deletion on the visual cycle, we analyzed cellular retinaldehyde–binding protein (CRALBP) levels in the inner retina using capillary electrophoresis. Interestingly, the GLAST-COX10^fl/fl^ retina showed an increase in MG-specific marker CRALBP (Figure 2B and Figure S4). Since MG function is fundamental to maintaining homeostasis within the retina, the effect of COX10 deletion on retinal function was investigated using electroretinography (ERG) analysis. Scotopic a-wave, corresponding to rod photoreceptor activity, had significantly reduced amplitude at various flash intensities in the GLAST-COX10^fl/fl^ retina (Figure 2C). Two-way ANOVA of a-wave amplitude indicated simple main effects for genotype (F(1,72) = 7.946; *p* = 0.0062) and light intensity (F(5,72) = 12.05; *p* < 0.0001). We also compared b-wave amplitude, which corresponds to ON-bipolar cell and MG responses, at different light intensities (Figure 2D). Two-way ANOVA simple main effects analysis showed that genotype (F(1,72) = 5.65; *p* = 0.02) and light intensity (F(5,72) = 14.43; *p* < 0.0001) each had statistically significant effects on the scotopic b-wave amplitude. Figure 2E shows representative scotopic ERG waveforms from dark-adapted control and Cox10 KO mice at 300 mcd.s/m^2^.

### 3.3. Oxidative Stress and MG Homeostasis

**Figure 3:**
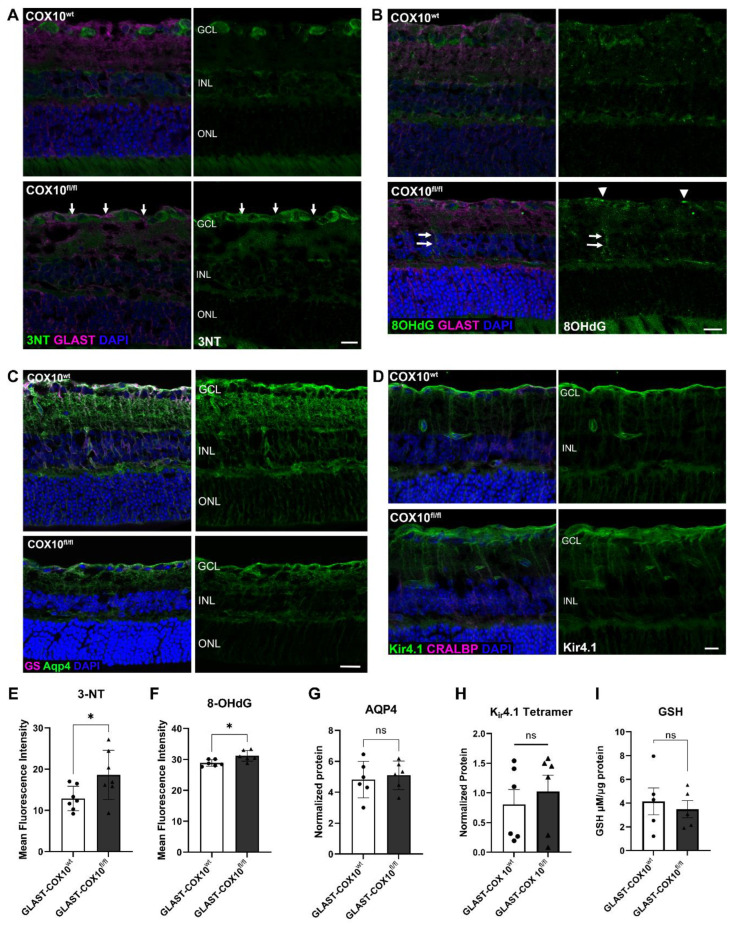
Expression of oxidative stress markers and glutathione in control and GLAST-COX10^fl/fl^ retinas. Immunolabeling for (**A**) 3-nitrotyrosine (3NT), a marker for protein nitrosylation shows greater 3NT label in the IPL of GLAST-COX10^fl/fl^ retina, and a shift of 3NT into the MG endfeet of GLAST-COX10^fl/fl^ retina from its primary location in retinal ganglion cells in the wildtype retina (white arrows). (**B**) Immunolabeling for 8OHdG, a marker of nucleic acid oxidation, illustrates an increase in labeling intensity in the ganglion cell layer (GCL) (white arrowheads), as well as in MG processes of GLAST-COX10^fl/fl^ retinas (white arrows). (**C**) Immunofluorescence of aquaporin-4 (Aqp4; green immunolabel) was not appreciably altered in the GLAST-COX10^fl/fl^ retina as compared to the wildtype. (**D**) Immunohistochemical analysis of K_ir_4.1 in retinal cross-sections showed no differences between control and GLAST-COX10^fl/fl^ retinas. (**E**) Quantification of 3-NT immunolabeling in retinal cross sections. GLAST-COX10^fl/fl^ retina had significantly higher 3-NT fluorescence compared to control retina (* *p* = 0.0419). (**F**) Quantification of 8OHdG immunolabeling in retinal cross sections. GLAST-COX10^fl/fl^ retina had significantly higher 8OHdG fluorescence compared to control retina (* *p* = 0.0177). (**G**) Quantification of Aqp4 protein from wildtype and GLAST-COX10^fl/fl^ retina. There was no statistical difference across the groups for Aqp4. (**H**) Quantification of K_ir_4.1 protein from wildtype and GLAST-COX10^fl/fl^ retina. There was no statistical difference across the groups for the tetrameric protein, or the trimeric protein, see Figure S6. (**I**) Total retinal glutathione (GSH) concentrations between wildtype and GLAST-COX10^fl/fl^ retinas were not statistically different. For panels E through I, error bars represent mean ± SEM GCL = ganglion cell layer; INL = inner nuclear layer; ONL = outer nuclear layer. Scale bars: 25 µm.

Oxidative stress depends on the balance of antioxidant synthesis and reactive oxygen species generation and can be determined by examining oxidative modifications of protein, DNA, and lipids. Immunolabeling for 3-nitrotyrosine (3NT), a marker for protein nitrosylation, shows greater 3NT label in the IPL of GLAST-COX10^fl/fl^ retina, and a shift of 3NT into the MG end feet of GLAST-COX10^fl/fl^ retina from its primary location in retinal ganglion cells in the wildtype retina (Figure 3A,E). Immunolabeling for 8OHdG, a marker of nucleic acid oxidation, was increased in the ganglion cell layer (GCL), as well as in MG processes of GLAST-COX10^fl/fl^ retinas (arrowheads and arrows, respectively; Figure 3B,F). Water balance in the retina is largely the purview of MG through the activity of their Aquaporin-4 (Aqp4) channels. We examined Aqp4 in the retinal cross sections, finding no gross alterations in the distribution of the protein in the GLAST-COX10^fl/fl^ retina (Figure 3C,G and Figure S5). To understand how loss of MG mitochondrial respiration affected extracellular potassium ion clearance properties of MG, we compared levels of K_ir_4.1 protein in control and GLAST-COX10^fl/fl^ mice. There was no difference in K_ir_4.1 protein levels between 3-month-old control and GLAST-COX10^fl/fl^ mice (Figure 3D,H and Figure S6). MG are the main source of glutathione (GSH) in the retina [20]; therefore, we examined GSH synthesis, finding no statistically significant differences in the total retinal glutathione (GSH) concentrations between wildtype and GLAST-COX10^fl/fl^ retinas (Figure 3I).

### 3.4. Changes in Retinal Metabolism

**Figure 4:**
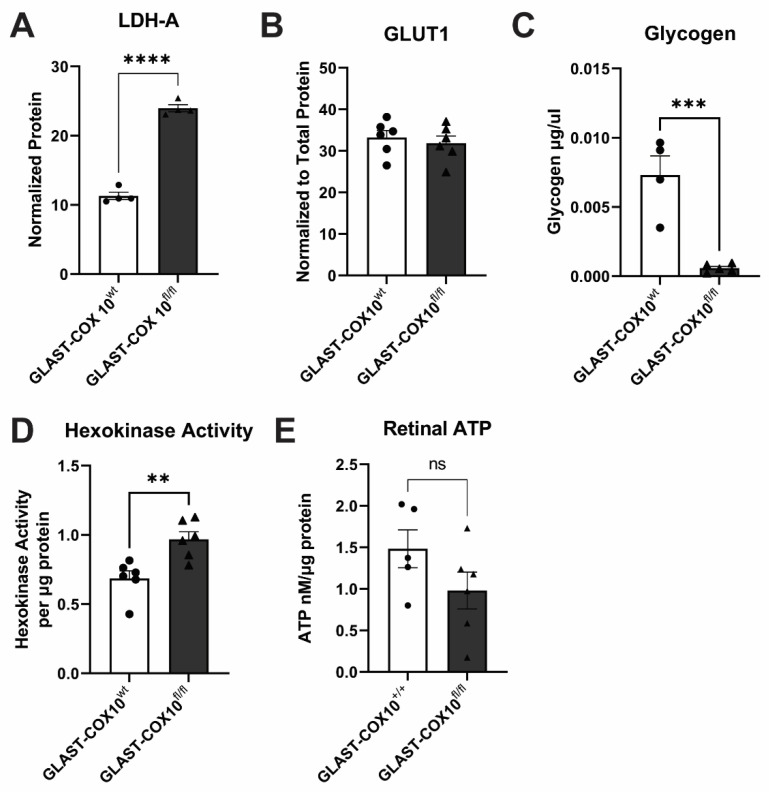
Bioenergetic changes in wildtype and GLAST-COX10^fl/fl^ mice. (**A**) Total LDH-A levels as measured by capillary electrophoresis are significantly increased in the GLAST-COX10^fl/fl^ retina as compared to wildtype control (two-tailed *t*-test, **** *p* < 0.0001). (**B**) Total GLUT1 protein levels are unchanged after destabilization of COXIV in MG as compared to wildtype control. (**C**) Total retina glycogen levels are significantly decreased in GLAST-COX 10^fl/fl^ retina as compared to wildtype control (two-tailed *t*-test, *** *p* = 0.0090). (**D**) Hexokinase activity is significantly increased in whole retinal lysates from GLAST-COX10^fl/fl^ mice as compared to wildtype control (two-tailed *t*-test, ** *p* = 0.0047). (**E**) Whole retinal-lysate ATP levels were not different between wildtype control and GLAST-COX10^fl/fl^ retinas. *n* = 5–6, mean + SEM.

To determine the impact of MG mitochondrial respiration loss on retinal energy metabolism, we assessed the levels and activity of glycolytic enzymes, glycogen, and total ATP. We measured lactate dehydrogenase-a (LDH-A) and glucose transporter-1 (GLUT1) protein using capillary electrophoresis. LDH-A is an isoform of the enzyme prominently expressed in retina [12], and GLUT1 is the glucose transporter found in MG, astrocytes, photoreceptors, and endothelial cells. GLAST-COX10^fl/fl^ mouse retina demonstrated a two-fold increase in LDH-A protein levels (Figure 4A and Figure S7), suggesting there is greater need for conversion of glycolysis-derived pyruvate to lactate (or lactate to pyruvate). We found no significant differences in GLUT1 levels in whole retinal lysates (Figure 4B and Figure S8), indicating no impetus for Cox10 knockout MG to promote GLUT1 upregulation as might be anticipated if needing more glucose for glycolysis. Glycogen concentration from total retinal lysate was significantly decreased in GLAST-COX10^fl/fl^ compared to wildtype controls, indicating usage of glucose as opposed to continued storage (Figure 4C). Hexokinase activity was significantly increased in mutant mice (Figure 4D), suggesting increased glycolysis with the destabilization of OXPHOS in MG. The higher hexokinase activity may account for the diminished glycogen observed. No significant changes were found in the expression of GLAST (Supplementary Data, Figure S3). Total ATP measured in retinal lysate showed no significant difference across the control and GLAST-COX10^fl/fl^ groups (Figure 4E). Collectively, these results demonstrate the promotion of glycolysis in the retina following COX10 knockout in MG.

### 3.5. COX10 Knockout and Ocular Hypertension

**Figure 5:**
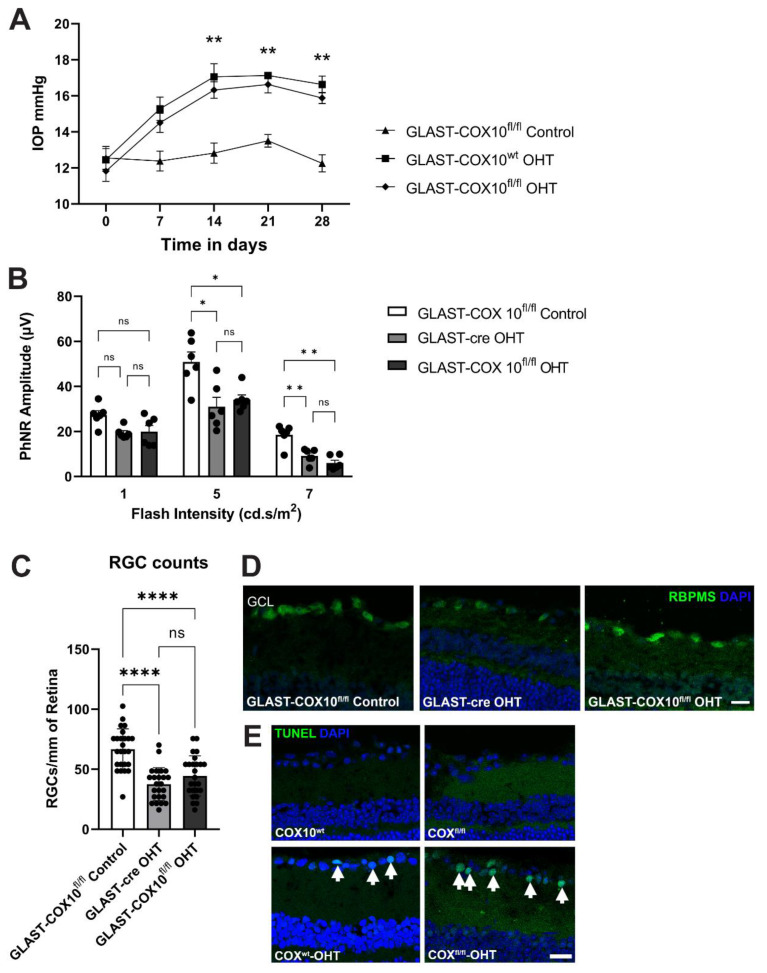
Effect of IOP elevation on photopic negative response in wildtype and GLAST-COX10^fl/fl^ mice. (**A**) Intraocular pressure was significantly higher in ocular hypertension (OHT) groups (GLAST-COX10^wt^ and GLAST-COX10^fl/fl^) than in GLAST-COX10^fl/fl^ control from 7d through 28d. Error bars represent mean ± SEM. (**B**) Photopic negative response (PhNR) mean amplitudes recorded from GLAST-COX10^fl/fl^ mice with and without OHT, plus wildtype (GLAST-cre) mice with OHT under light-adapted conditions showed no differences among groups at 1cd.s/m^2^. At 5cd.s/m^2^, both OHT groups had significantly lower PhNR than the knockout without OHT (* *p* < 0.05). A further reduction of PhNR amplitude was observed at 7cd.s/m^2^, with both OHT groups having significantly reduced PhNR as compared to the GLAST-COX10^fl/fl^ mice without OHT (** *p* < 0.01). Values are ± SEM; 6–8 mice per group. * *p* < 0.05, ** *p* < 0.01. (**C**) RGC quantification from retinal cross-sections show significantly higher RGC number in GLAST-COX10^fl/fl^ control retina as compared to both GLAST-cre with OHT (**** *p* < 0.0001) and GLAST-COX10^fl/fl^ with OHT (**** *p* < 0.0001). Error bars represent mean ± SEM. (**D**) Representative immunofluorescence images of retinas from each experimental group that were used for RGC quantification in (**C**). RBPMS (green) was used as an RGC marker. (**E**) TUNEL labeling to identify apoptotic cells (white arrows) in retina showed apoptotic cells in the COX10^wt^ and COX10^fl/fl^ groups with OHT only. Scale bars = 25 µm.

Metabolic dysfunction has been reported in RGCs from glaucoma patients and in animal models [2,4,5,40,41,42], and we have reported the sensitivity of MG to OHT-associated hypoxia [31,32], so we examined the impact of MG-specific COX10 knockout on RGC survival following 4 weeks of ocular hypertension. Significant IOP elevation was observed 7 days after microbead injection and lasted for 3 additional weeks (Figure 5A); there was no difference in IOP between the groups that underwent ocular hypertension (OHT). We measured the photopic negative response (PhNR) to evaluate RGC function after OHT. The average PhNR amplitudes of OHT eyes were lower compared to the untreated GLAST-COX10^fl/fl^ at all light intensities. The difference between the average PhNR amplitude of OHT eyes and untreated controls were statistically significant, except at 1cd.s/m^2^ (Figure 5B). Disrupting OXPHOS in MG did not further exacerbate the PhNR after OHT. There were significantly fewer RGCs in OHT mice compared to control mice (Figure 5C). Similarly to PhNR, Cox10 knockout did not compound RGC loss after OHT; the GLAST-COX10^fl/fl^ retinas with OHT had similar RGC survival as control mice with OHT. Figure 5D shows representative immunofluorescence images for retina cross-sections used for RGC quantification. TUNEL labeling was performed on retinas for wildtype control and OHT, and GLAST-COX10^fl/fl^ with or without OHT (Figure 5E). Apoptotic cells were only observed in the retinal cross-sections from mice having undergone OHT. The ganglion cell layer (GCL) was shown because no apoptotic cells were observed in any other retinal layer. There was no evidence of MG death in the COX10^fl/fl^ mice, with or without OHT.

## 4. Discussion

Our goal with this work was two-fold, to understand why MG are sensitive to hypoxia as shown in our previous work [31,32], and to shed additional light on fundamental MG metabolism. By knocking out COX10, a heme farnesyltransferase, in GLAST-expressing cells, we sought to destabilize OXPHOS in the retina and evaluate the impact on MG morphology and function. Knocking out COX10 to disrupt OXPHOS is a strategy previously used to explore the role of mitochondrial respiration in specific cell types in vivo [10,11]. The absence of COX10 prevents the assembly and stabilization of cytochrome-c oxidase (COX), the final enzyme (complex IV) of the electron transport chain [38]. As anticipated, deleting COX10 led to significantly lower levels of COXIV protein in primary MG cell lysates and MG end feet. Moreover, increased SDH staining indicating COX deficient cells was higher in COX10 knockout retinas than controls. Through DNA, protein, and enzymatic analysis, we demonstrated that GLAST-cre-driven elimination of COX10 occurred in the retina, and it resulted in reduced COXIV protein and activity.

Given that disruption of OXPHOS in MG may induce MG dysfunction and retinal damage, we analyzed GFAP protein expression and visual function. Increased expression of GFAP by MG in response to stress has been previously reported [43,44,45]. Interestingly, GFAP expression in COX10 knockout retina was found to be limited to the nerve fiber layer, similar to the control retina. This observation is consistent with previous reports that selectively blocking OXPHOS in glial cells in the CNS using COX10 mice does not induce reactive gliosis [10,11]. Next, we tested whether diminished MG mitochondrial respiration affected visual function. Our results showed lower a- and b-wave amplitudes from ERG recordings in COX10 knockout mice. We evaluated scotopic ERG, for which the a-wave corresponds to rod photoreceptor activity. Investigation of the metabolic coupling of MG and photoreceptors suggests that lactate from the photoreceptors is provided to MG for their use in OXPHOS [13]. By destabilizing COXIV in MG, we may have compromised the uptake of lactate from photoreceptors, thereby negatively impacting rod photoreceptor activity as reported by the decline in a-wave amplitude in the COX10 knockout mice. Scotopic b-wave is the activity output of ON bipolar cells, and to a lesser degree, MG. We also observed a b-wave amplitude decrease in the COX10 knockout mice, which supports the idea that the MG physiology is impaired because ON bipolar cells would not be expected to be stimulated under scotopic conditions, and ON bipolar cells have not been found to contact rod photoreceptors in the mouse retina [46]. Interestingly, CRALBP was significantly upregulated in the GLAST-COX10^fl/fl^ retina. CRALBP exists in both the retinal pigment epithelium and the MG. In MG, CRALBP shuttles 11-cis retinaldehyde to cone photoreceptors as part of the non-canonical cone visual cycle [47]. Our retinal isolation technique does not collect the RPE, so the CRALBP increase should be representative of MG change, suggesting that altering the MG metabolism may have promoted the cone visual cycle. This could be an informative direction for future investigation.

K_ir_4.1 is critical to MG management of K^+^ currents and extracellular glutamate concentration in the retina [24,48]. Impairments in ERG response have been observed in K_ir_4.1^−/−^ mice [48,49], so we examined the effect of selective inhibition of OXPHOS on K_ir_4.1 expression. IHC analysis and capillary electrophoresis revealed K_ir_4.1 protein levels unchanged in KO retinas compared to the control, indicating that these vital roles of MG are likely unchanged as a result of COX10 knockout. The glutamate–glutamine cycle can be an important source for TCA cycle intermediates, so the potential for the maintenance of glutamate concentrations through stable K_ir_4.1 and GLAST protein levels offers additional means by which MG can manage their metabolism in light of the COX10 knockout.

MG are the major source of glutathione, the main antioxidant in the retina [20,26]. Previous research reported decreased glutamate uptake by cultured MG with mitochondrial dysfunction [34]. As glutamate is a primary source for GSH synthesis [9], we examined glutathione levels and oxidative stress. Immunolabeling showed increased levels of 3-NT, a result of the free radical peroxynitrite (formed from nitric oxide and mitochondrial superoxide) that attacks proteins, in MG endfeet of COX10 knockout retinas. The immunofluorescence of 8-OHdG, a result of the reactive oxygen species attack of nucleic acid, was also increased in MG basal processes and the endfeet of COX10 knockout retinas. Evidence of oxidative stress particularly in MG endfeet is likely a reflection of the high concentration of mitochondria there [50]. Total glutathione levels remained unchanged after COX10 knockout, suggesting the 3-NT- and 8-OHdG-associated oxidative stress is likely due to mitochondrial dysfunction in MG. Relatedly, mitochondrial dysfunction and oxidative stress can cause MG swelling in diabetic retina [6]. AQP4 channels, mainly expressed by MG, play important roles in regulating water homeostasis in the retina. We observed little change in AQP4 levels in COX10 knockout mice compared to control; additionally, there was no evidence of retina swelling. Knocking out Aqp4 in MG led to hyperactive scotopic ERG in mouse retina [51]; given our observation of decreased scotopic ERG with COX10 knockout, stable Aqp4 protein in the COX10 knockout retina is consistent with our physiology recordings.

The loss of mitochondrial respiration in the GLAST-COX10^fl/fl^ retina predicts that glycolysis, or alternative pathways, would be elevated to meet MG ATP demand. LDH-A is the primary lactate dehydrogenase enzyme expressed in the retina [36]. It catalyzes the conversion of pyruvate derived from glycolysis into lactate, and vice versa [36]. Though LDH-A and hexokinase have been described as deficient in MG [13,52], we detected significant increases in total LDH-A protein levels and hexokinase activity in retinas from COX10 KO mice compared to controls. We were analyzing whole retina lysate, so we cannot ascribe these changes solely to MG. However, we can conclude that destabilizing OXPHOS in MG does promote glycolysis more globally in the retina. Photoreceptors, as the major consumers of glucose, are candidates for a major contribution to elevated hexokinase activity. It may be the case that the metabolic coupling of photoreceptors to MG with compromised OXPHOS could result in a compensatory increase in photoreceptor glycolysis. However, our ERG data indicating reduced photoreceptor electrical activity argues against greater glycolysis in those cells. Alternatively, MG may have a mechanism by which they use glycolysis as an adjunct to diminished OXPHOS to meet their energy needs, despite evidence to the contrary [12,13]. This would be a key hypothesis for future study.

GLUT1 is the main glucose transporter expressed by MG [13]. Despite the increased hexokinase activity, we did not find significant changes in GLUT1 protein expression or total ATP in COX10 KO retinas. Stable GLUT1 in the retina despite diminished OXPHOS in MG supports a non-MG source for the increased hexokinase activity, perhaps the photoreceptors. There was, however, a significant decrease in glycogen content. Glycogen, the storage form for glucose, is found in MG and astrocytes in the retina [53]. Low glycogen with COX10 knockout could indicate that internal glycogen stores from MG are being broken down to produce glucose for the increased glycolysis [54,55,56,57]. The destination for the glucose liberated from these glycogen stores is unknown. Ultimately, these data suggest increased glycolysis in the retina may be compensating for the diminished ATP generation from the loss OXPHOS in MG, raising the possibility that MG-specific OXPHOS is important in generating ATP in the retina. Maintenance of ATP levels despite the lack of fully functional MG OXPHOS shows that the retina was nevertheless able to meet its energy needs, and likely did so through glycolysis. These data underscore the versatility of this system of retinal cells that are metabolically linked.

Finally, we directly tested the importance of MG OXPHOS to retinal ganglion cell survival after ocular hypertension (OHT). Our previous work showed that OHT is associated with hypoxia and at least intermittent limits on glucose availability [31,32]. MG respond to OHT with a dramatic upregulation of HIF-1α [32]. As a result, we hypothesized that RGC loss in COX10 knockout retina would be greater than the control retina after OHT. OHT resulted in significantly lower PhNR amplitude and RGC numbers, regardless of genotype (COX10 knockout or wildtype). Surprisingly, we observed no exacerbation of OHT-associated pathology as a result of COX10 knockout in MG. Despite the altered bioenergetics in COX10 knockout retina compared to the wildtype, inhibiting MG mitochondrial respiration did not increase RGC dysfunction nor accelerate RGC loss.

## 5. Conclusions

In summary, our data show that altering OXPHOS in MG shifts retinal metabolism towards increased glycolysis and compromises photoreceptor and MG physiological response. The COX10 knockout increased oxidative stress in MG but did not overtly impact MG water homeostasis and K^+^ management function. The increased CRALBP and altered scotopic a-wave indicate that OXPHOS in COX10 knockout MG affected the photoreceptor function, underscoring the close metabolic relationship these cells share. Finding RGCs not additionally damaged by the metabolic shift in COX10 knockout MG suggest compensatory mechanisms in the retinal metabolic landscape that support continued function in the face of significant stressors.

## Figures and Tables

**Table 1:** Antibodies used in immunofluorescence and capillary electrophoresis.

Antigen	Species	Manufacturer	Catalog Number	Dilution
AQP4	Rabbit	Abcam	ab61392	1:250 IF; 1:50 CE
COX 10	Rabbit	Novus Biologicals	NBP1-59554	1:100 IF
COX IV	Rabbit	Cell Signaling	4850	1:100 IF; 1:25 CE
CRALBP	Mouse	Novus Biologicals	NB 100-74392	1:200; 1:50 CE
GFAP	Goat	Abcam	ab53554	1:500
GLAST	Rabbit	Abcam	ab416	1:200 IF; 1:50 CE
Glutamine Synthetase	Mouse	Santa Cruz	SC-74430	1:150
MTCO1	Mouse	Invitrogen	459600	1:100
RBPMS	Rabbit	Genetex	118619	1:300
Vimentin	Chicken	Novus Biologicals	NB300-233	1:250
GLUT1	Rabbit	Novus Biologicals	NB110-39113	1:50 CE
LDH-A	Rabbit	Novus Biologicals	NBP1-48336	1:50 CE
Kir4.1	Rabbit	ProteinTech	12503-1-AP	1:50 CE
8-Hydroxy-deoxy-guanosine (8OHdG)	Rabbit	QED Bioscience	12501	1:200
3-Nitrotyrosine	Mouse	Abcam	ab61392	1:200

IF = immunofluorescence; CE = capillary electrophoresis.

**Table 2:** Biochemical assay kits.

Parameter	Manufacturer	Catalog Number
Glycogen	Millipore-Sigma	MAK016-1KT
Glutathione	Cayman Chemical	703002
Hexokinase	Millipore-Sigma	MAK091-1KT
ATP	Invitrogen	A22066
TUNEL	Promega	G7130

## Data Availability

Data supporting reported results are contained in the figures and Supplementary Data of the paper.

## Supplementary Materials

The following supporting information can be downloaded at: https://www.mdpi.com/article/10.3390/cells11233756/s1. Figure S1: Further characterization of the GLAST-COX10^fl/fl^ retina; Figure S2: COXIV protein capillary electrophoresis in Müller glia lysates; Figure S3: GLAST capillary electrophoresis using retinal lysates; Figure S4: CRALBP capillary electrophoresis using retinal lysates; Figure S5: AQP4 capillary electrophoresis using retinal lysates; Figure S6: K_ir_4.1 capillary electrophoresis using retinal lysates; Figure S7: LDH-A capillary electrophoresis using retinal lysates; Figure S8: GLUT1 capillary electrophoresis using retinal lysates. References [58,59,60] are cited in the Supplementary materials.
