# Supplementary material for: Destabilizing COXIV in Müller Glia Increases Retinal Glycolysis and Alters Scotopic Electroretinogram
Source: Cells. 2022 Nov 24;11(23):3756. doi: 10.3390/cells11233756 (PMC9737073; doi:10.3390/cells11233756)
Supplement: Supplementary file 1 [file cells-11-03756-s001.zip › cells-1998852-supplementary.pdf]

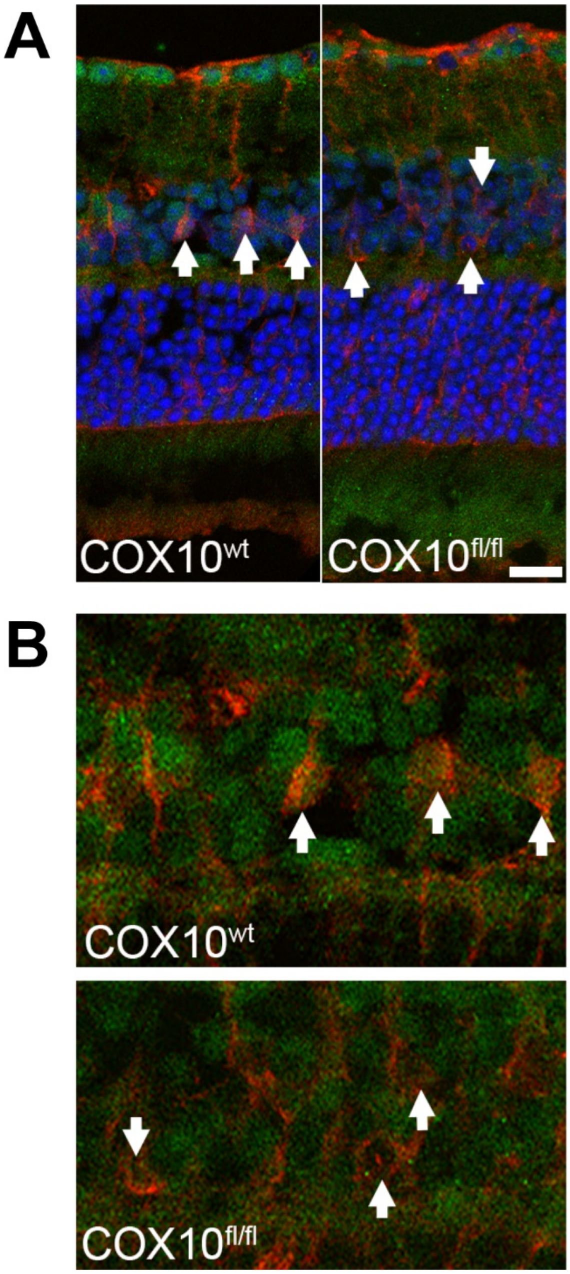

Figure S1. Further characterization of the GLAST-COX10$^{fl/fl}$ retina. A) Immunofluorescence of retina from control (COX10^{wt}) and knockout (GLAST-COX10$^{fl/fl}$) mouse retina, labeled with antibodies against COX10 (green), CRALBP (red), and DAPI (blue). White arrows indicate Müller glia cell bodies. B) Higher magnification views of each inner nuclear layer (below the top panels) show COX10 colocalization with CRALBP (yellow) in COX10^{wt}, but not COX10$^{fl/fl}$ Müller glia. Scale bar=25µm

Figure S2. COXIV Capillary Electrophoresis in Müller glia cell lysates

A)

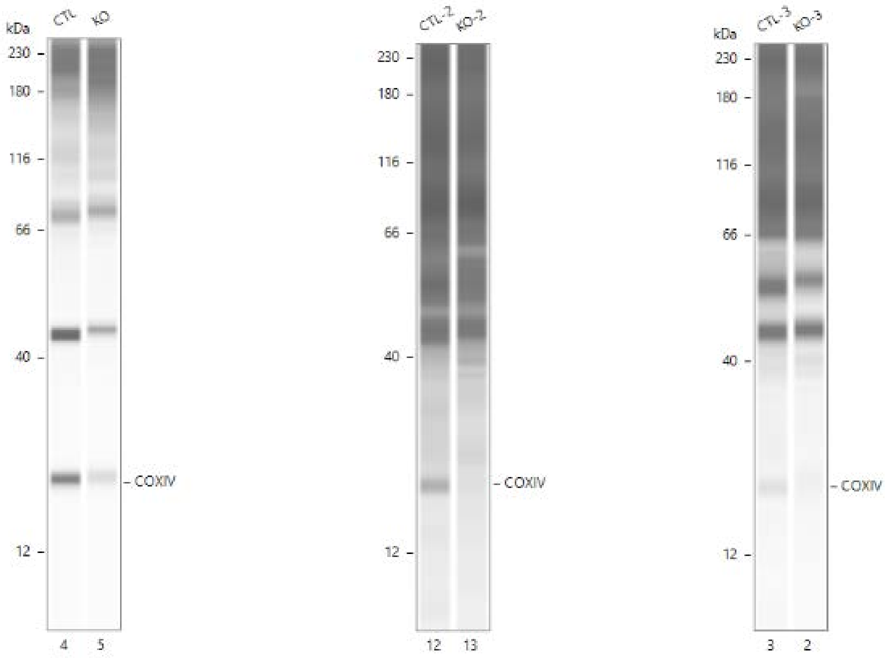

B)

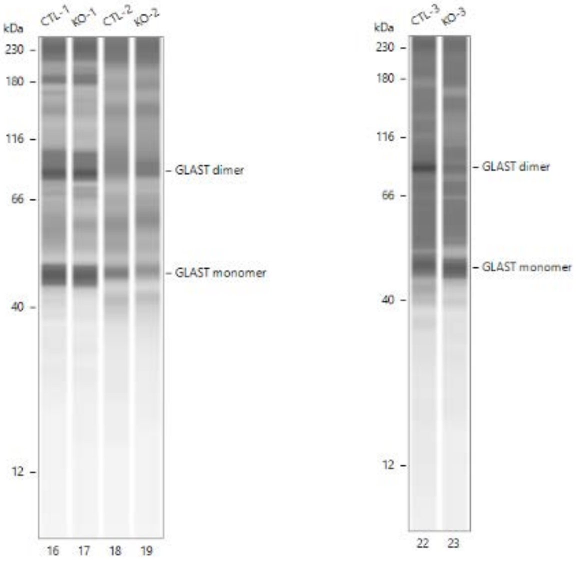

C) COXIV area/ GLAST area

Control	Knockout
0.607672	0.45262
1.12948	0.338296
0.801173	0.466564

Figure S2. Capillary electrophoresis output used in **Figure 1B**. Lysates from primary Müller glia cultured for 8 weeks from control (GLAST-COX10^{wt}) and knockout (GLAST-COX10$^{fl/fl}$) mouse retina were probed for A) COXIV (22 kDa) and B) GLAST (monomer 49 kDa and dimer 82 kDa). The lanes shown are representations of the capillary electrophoresis chemiluminescence output traces; they are not traditional western blots. For Figure 1B, the COXIV protein was normalized to GLAST protein (49 kDa) because GLAST protein is a Müller glial marker and has been shown to not vary across these samples (see Figure S3). The table in C) shows the quantitative values used for the bar chart in Figure 1B.

Figure S3. GLAST capillary electrophoresis using retinal lysates

A)

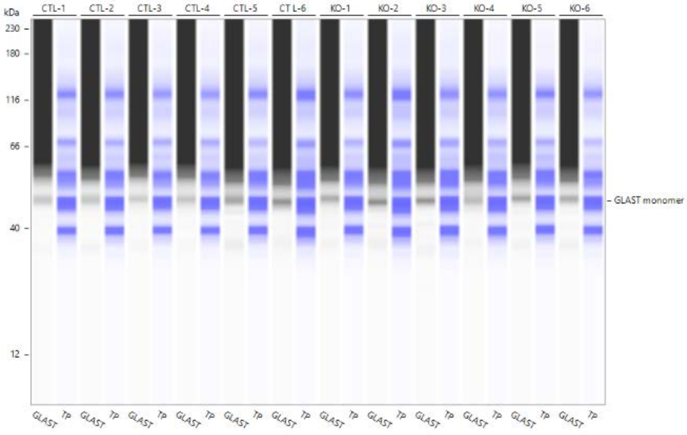

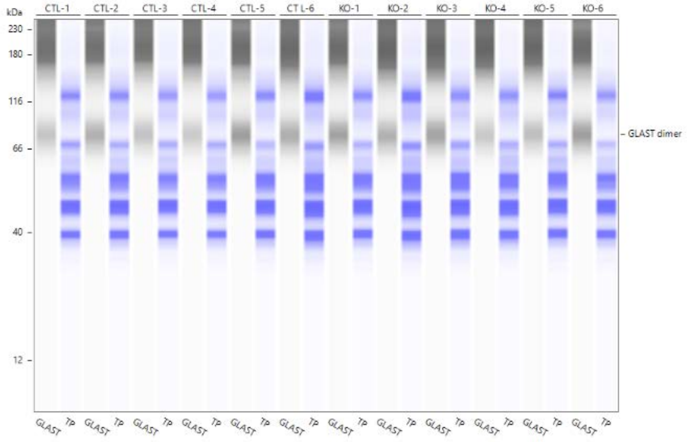

B)

GLAST monomer area/Total protein area

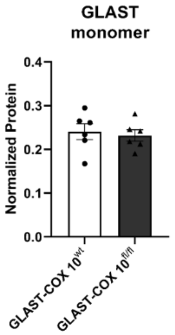

Control	Knockout
0.220722	0.281372
0.261122	0.246282
0.264832	0.242155
0.167202	0.212588
0.233802	0.190424
0.294734	0.218044

GLAST dimer area/Total protein area

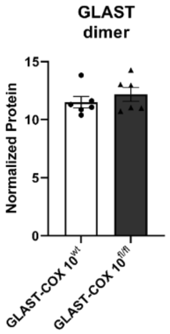

Control	Knockout
10.3919	13.04605
11.61767	10.99071
11.06842	12.97015
10.86377	10.72524
13.82779	11.04105
11.30097	14.28124

Figure S3. Capillary electrophoresis output from the Jess Protein Simple instrument for GLAST in control (GLAST-COX10^{wt}) and knockout (GLAST-COX10$^{fl/fl}$) mouse retina. A) These lane views show the antibody probe for each sample on the left (black and white), while the total protein (TP) lane for the sample is shown to the right for each sample pair (blue). The first set of lanes (left) are optimized to visualize the GLAST monomer (49 kDa), while the lanes on the right are optimized to visualize the GLAST dimer (82 kDa). The band representing the interaction of the primary antibody with the lysate (labeled GLAST monomer or dimer) is quantified and divided by the quantification of the total bands shown in the total protein (blue) lane; this is the normalized protein to which we refer in the figures showing all quantified protein. B) GLAST protein (monomer or dimer) does not vary across GLAST-COX10^{wt} (control) and GLAST-COX10$^{fl/fl}$ (knockout) retinal lysate (bar chart data). The values shown in each bar chart are listed in the accompanying table to the right of each chart. The stability of the GLAST protein in control and knockout retina ensured that GLAST-promoted cre-recombinase did not differentially affect control and knockout retinas.

Figure S4. CRALBP capillary electrophoresis using retinal lysates

A)

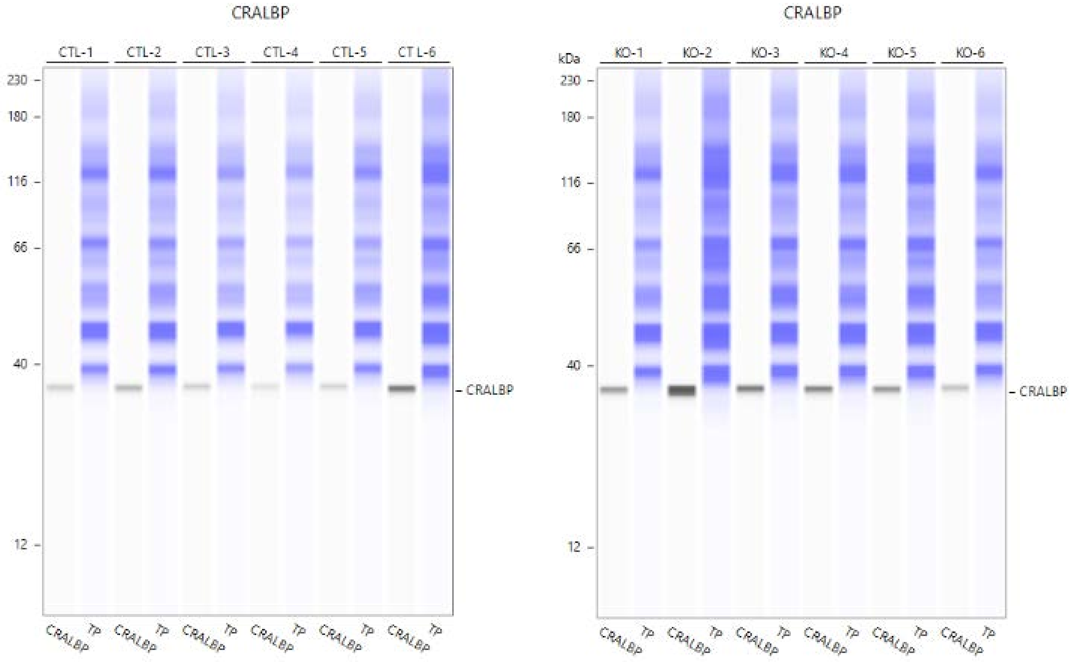

B) CRALBP area /Total Protein area

Control	Knockout
0.098466	0.155119
0.126674	0.276708
0.114365	0.167177
0.129657	0.156102
0.099991	0.13541
0.141806	0.113166

Figure S4. Capillary electrophoresis output used for quantitative data in **Figure 2B**. Lysates from control (GLAST-COX10^{wt}) and knockout (GLAST-COX10$^{fl/fl}$) mouse retina were probed for CRALBP (36 kDa) and total protein (TP). These lane views show the antibody probe for each sample on the left (black and white), while the total protein (TP) lane for the sample is shown to the right for each sample pair (blue). The table in B) shows the quantitative values (CRALBP band values divided by TP values) used for the bar chart in Figure 2B.

Figure S5. AQP4 capillary electrophoresis using retinal lysates

A)

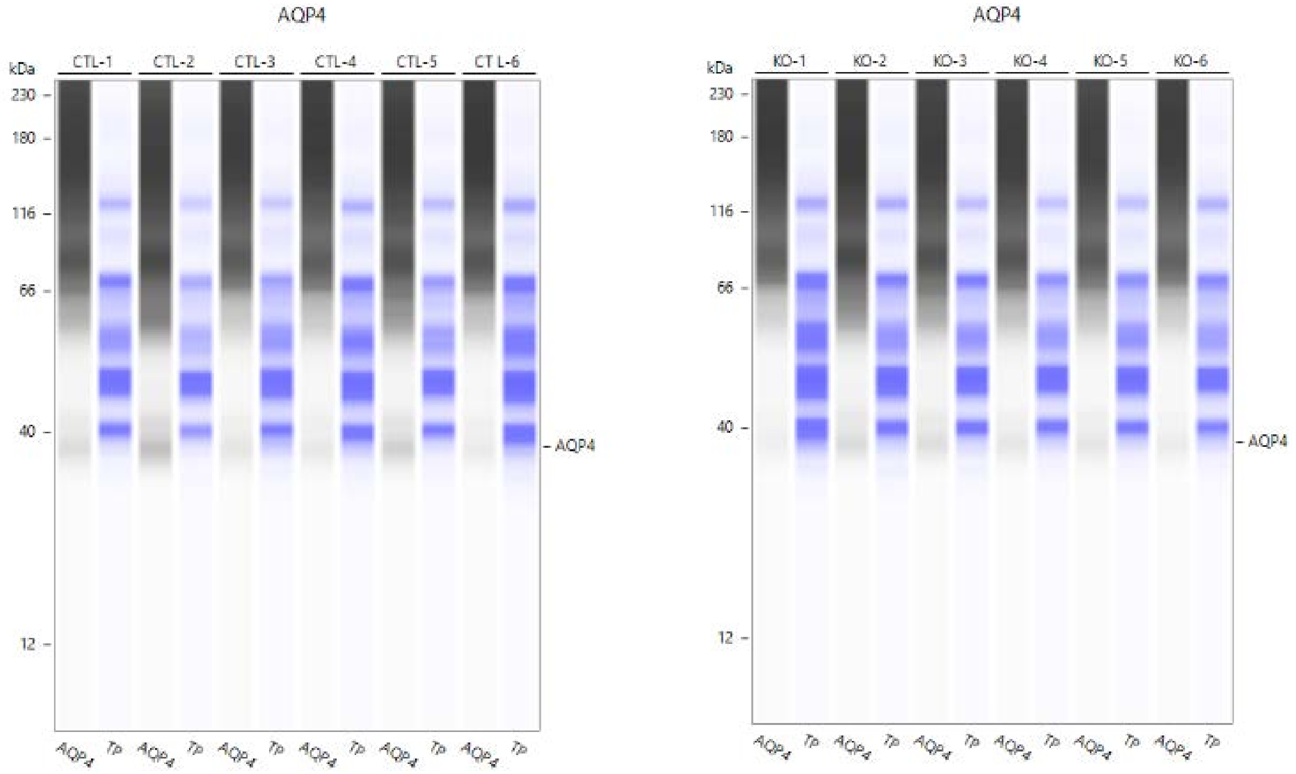

B)

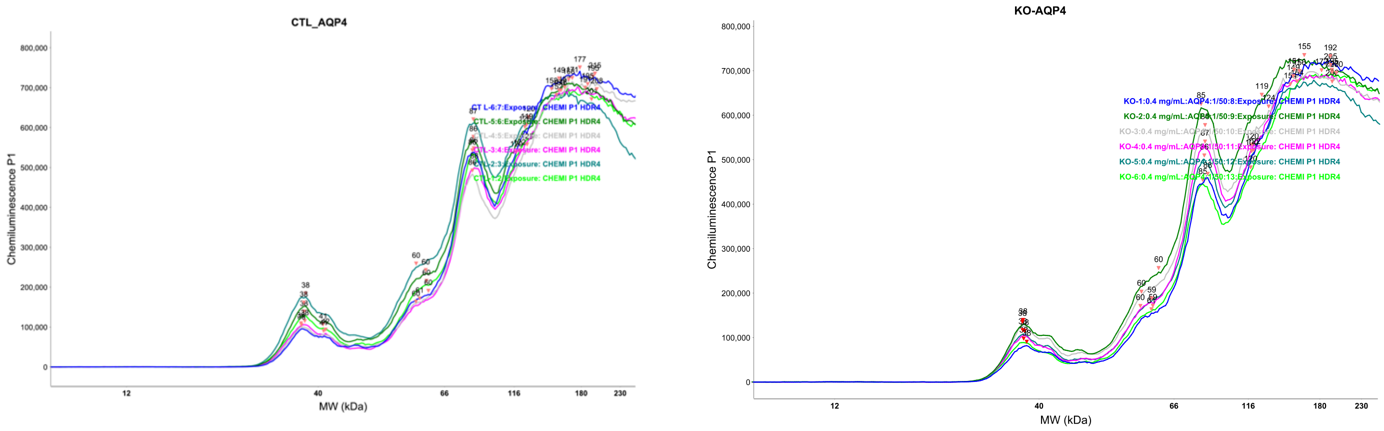

C)

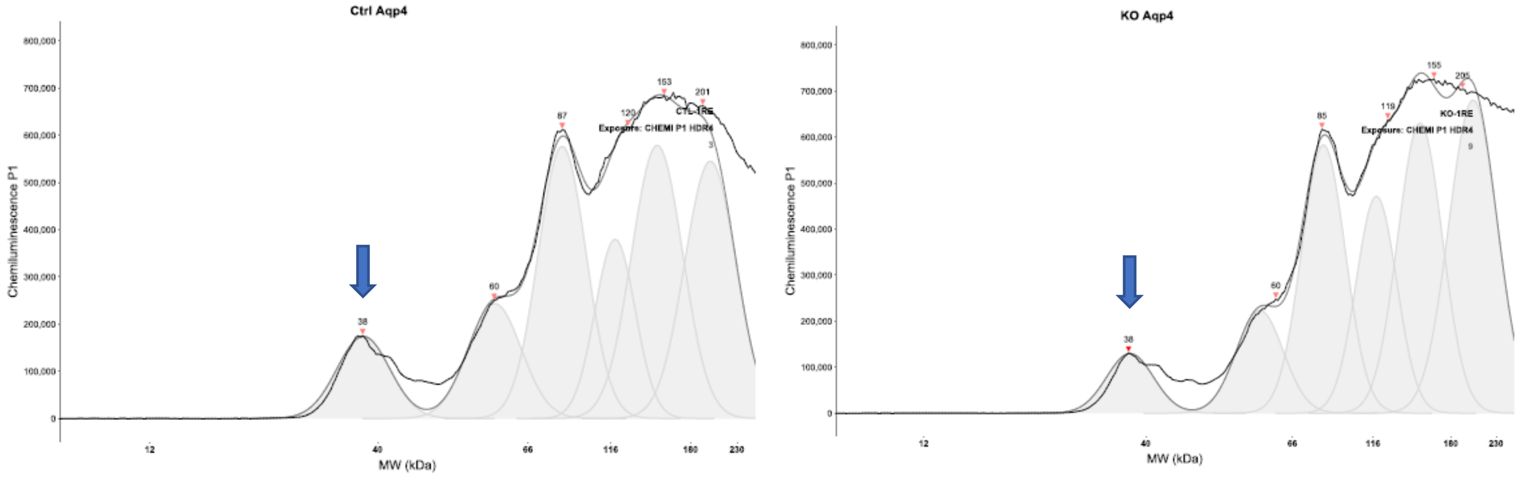

D) AQP4 area/ Total protein area

Control	Knockout
4.527658	3.62973
6.450962	6.180715
5.64348	5.801299
3.002298	5.153153
4.975558	5.331916
4.308102	4.469648

Figure S5. Capillary electrophoresis output used to generate data for bar chart in **Figure 3G.** Lysates from control (GLAST-COX10^{wt}) and knockout (GLAST-COX10$^{fl/fl}$) mouse retina were probed for AQP4 (38 kDa) and total protein (TP). These lane views show the antibody probe for each sample on the left (black and white), while the total protein (TP) lane for the sample is shown to the right for each sample pair (blue). The lane views are representations of chemiluminescence plots as shown in B). In the plots, each colored line corresponds to the chemiluminescence plot for one sample. We are showing the chemiluminescent plots for Aqp4 because the lane bands are not as distinct as those shown for the previously analyzed proteins. The plots indicate that a clear peak, corresponding to the Aqp4 monomer, is discernible at 38kDa. Higher molecular weight bands are oligomeric (dimers, trimers, and tetramer) versions of Aqp4 (Nagelhus et al., 1998; Neely et al., 1999). C) The area under the curve at 38kDa is the area of the quantified Aqp4 protein that will be divided by the area under the curve of the total protein (TP). The plots in C show a control (left) and a knockout (right) retinal lysate sample, with the blue arrow indicating the Aqp4 38kDa peak and corresponding area under the curve (gray) that was used for quantification. The table in D) shows the quantitative values obtained from the chemiluminescent plots in B) that were normalized to TP and used for the bar chart in Figure 3G.

Figure S6. Kir4.1 capillary electrophoresis using retinal lysates

A)

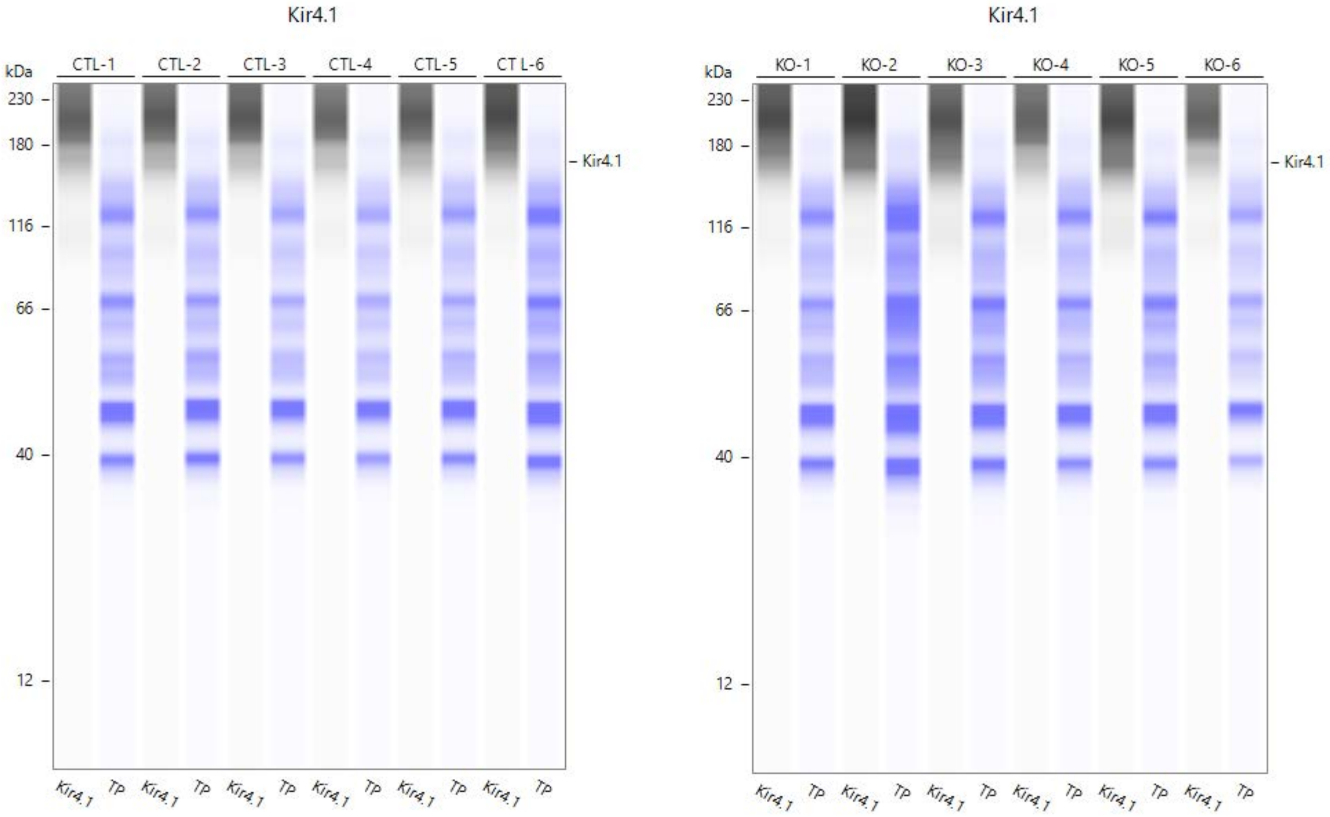

B)

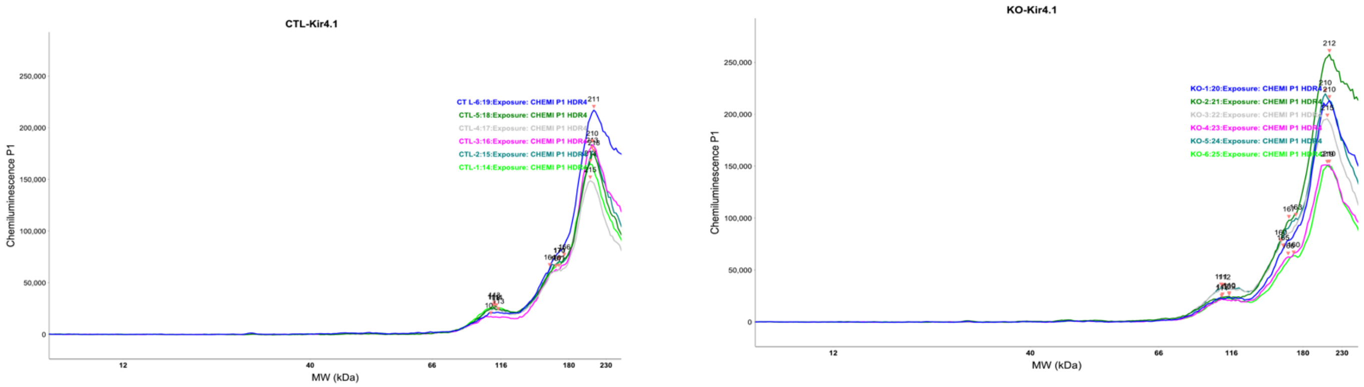

C) Control Example of K_{ir}4.1 Trimer (red arrow) and Tetramer (blue arrow) area under the curve (left); Knockout on (right)

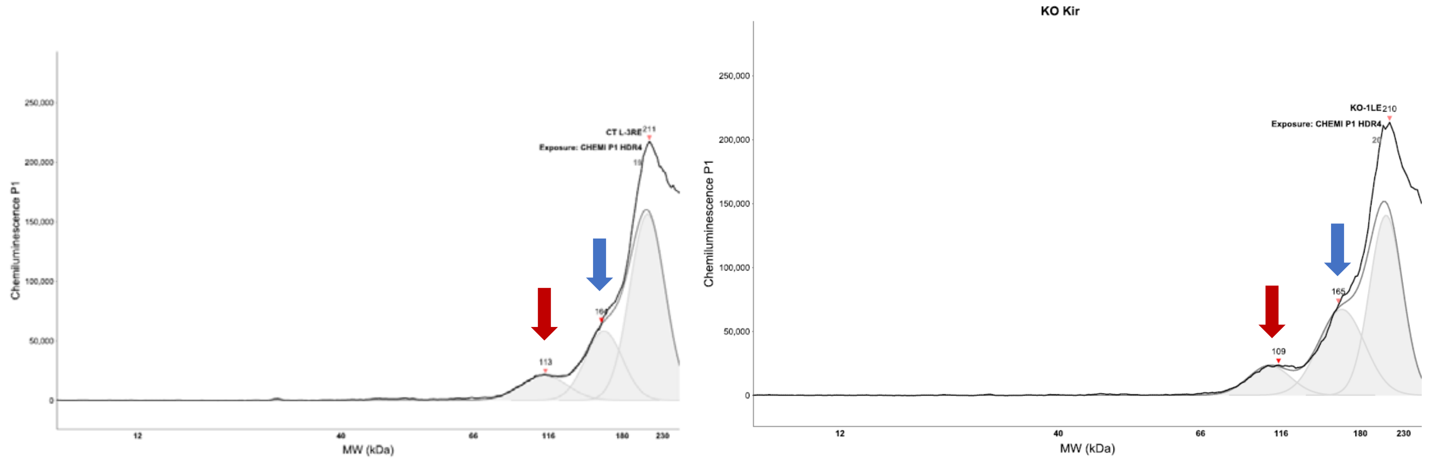

D) K_{ir}4.1 area/Total protein area for Tetramer and Trimer channels

Tetramer (165 kDa)	
**Control**	**Knockout**
0.19486	1.579808
1.541356	1.450062
1.407176	0.077042
0.266673	1.264861
0.314941	1.497576
1.106042	0.286353

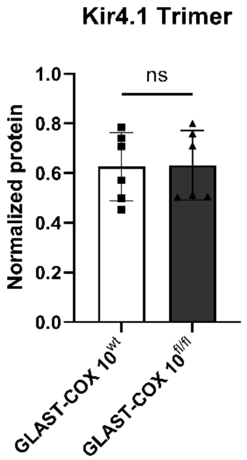

Trimer (113kDa)	
**Control**	**Knockout**
0.785796	0.504088
0.570915	0.511876
0.452026	0.800492
0.740375	0.505597
0.707577	0.760246
0.499224	0.711293

Figure S6. Capillary electrophoresis output used to generate bar chart shown in **Figure 3H.** Lysates from control (GLAST-COX10^{wt}) and knockout (GLAST-COX10$^{fl/fl}$) mouse retina were probed for K_{ir}4.1 (165 kDa) and total protein (TP). K_{ir}4.1 monomers are 42kDa, but this ion channel exists in heteromers and tetramers (Brasko, et al, 2017). Our protein analysis indicates the trimer (113 kDa) and tetramer (165kDa) are the predominant form we observed in mouse retina lysates. The lane views (A) show the antibody probe for each sample on the left (black and white), while the total protein (TP) lane for the sample is shown to the right for each sample pair (blue). B) Plots of the capillary electrophoresis chemiluminescence output show the range of sample output for the control (left) and knockout (right) retinal lysate protein. C) Plots of the capillary electrophoresis chemiluminescence output show the area under the curve (gray) used for the quantification of K_{ir}4.1 protein in one control (left) and one knockout (right) retina; peaks at 113 (red arrow) and 165 kDa (blue arrow) were used for K_{ir}4.1 quantification. D) The top table "Tetramer" contains the quantitative values derived from dividing the area under the curve for 165kDa K_{ir}4.1 by the TP area under the curve; these values were used for the bar chart in Figure 3H. The bottom table "Trimer" contains the quantitative values derived from dividing the area under the curve for 113kDa K_{ir}4.1 by the TP area under the curve; these values were used for the bar chart to the right. Similarly to the K_{ir}4.1 tetramers, there was no significant difference in the K_{ir}4.1 trimer protein in control versus knockout. Since neither K_{ir}4.1 oligomer differed between control and knockout mouse retina, only the tetramer data (165kDa) is shown in Figure 3H.

Figure S7. LDH-A capillary electrophoresis using retinal lysates

A)

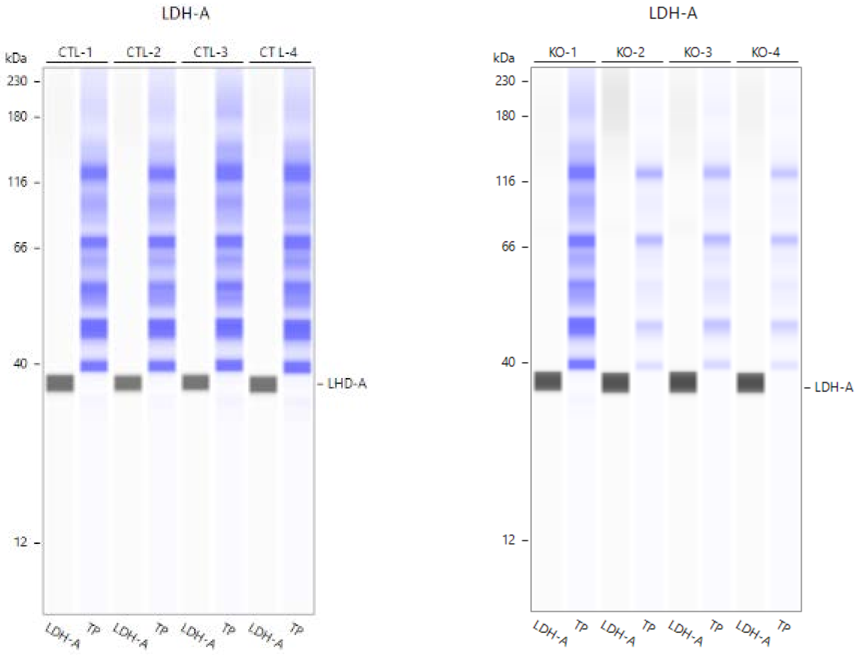

B) LDH-A area/Total protein area

Control	Knockout
12.87742	23.73152
10.93047	23.64766
10.50162	25.43855
10.93718	23.08145

Figure S7. Capillary electrophoresis output used to generate quantitative data shown in **Figure 4A.** Lysates from control (GLAST-COX10^{wt}) and knockout (GLAST-COX10$^{fl/fl}$) mouse retina were probed for LDH-A (36 kDa) and total protein (TP). These lane views show the antibody probe for each sample on the left (black and white), while the total protein (TP) lane for the sample is shown to the right for each sample pair (blue). The table in B) shows the quantitative values derived from the area under the curve of the chemiluminescent plots corresponding to the ratio of LDH-A to TP that were used for the bar chart in Figure 4A.

Figure S8. GLUT1 capillary electrophoresis using retinal lysates

A)

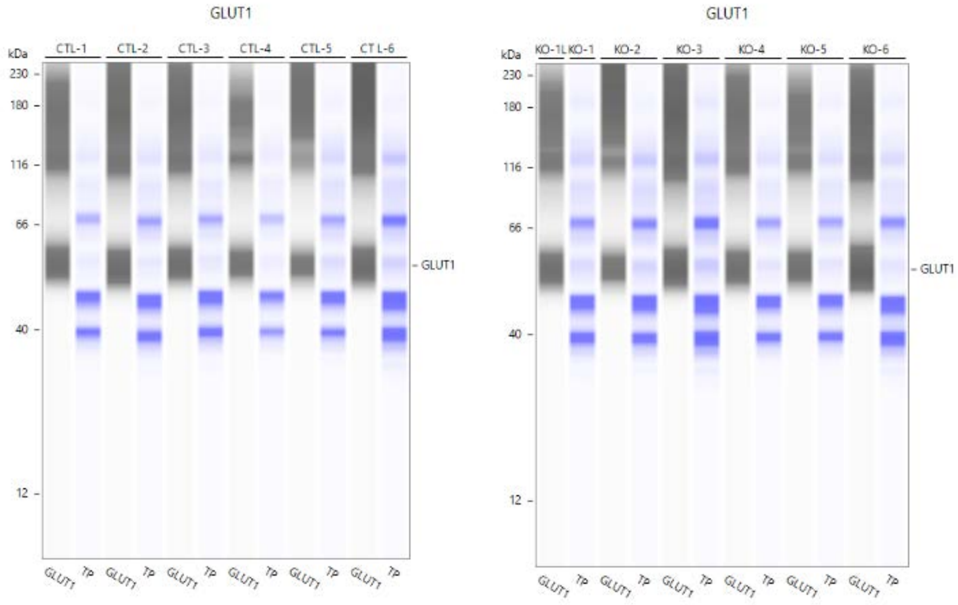

B) GLUT1 area/Total protein area

Control	Knockout
38.17839	29.84916
34.73915	24.86684
33.88787	31.09962
35.75361	35.17888
26.52747	33.06945
30.49543	37.02675

Figure S8. Capillary electrophoresis output used to generate quantitative data shown in **Figure 4B.** Lysates from control (GLAST-COX10^{wt}) and knockout (GLAST-COX10$^{fl/fl}$) mouse retina were probed for GLUT1 (55 kDa) and total protein (TP). These lane views show the antibody probe for each sample on the left (black and white), while the total protein (TP) lane for the sample is shown to the right for each sample pair (blue). The table in B) shows the quantitative values derived from the area under the curve of the chemiluminescent plots corresponding to the ratio of GLUT1 to TP used for the bar chart in Figure 4B.

References for Supplementary Data Figure Legends

Brasko C, Hawkins V, Chacon De La Rocha I, Butt AM. (2017) “Expression of Kir4.1 and Kir5.1 inwardly rectifying potassium channels in oligodendrocytes, the myelinating cells of the CNS.” *Brain Struct Funct* 222:41-59.

Nagelhus EA, Veruki ML, Torp R, Haug FM, Laake JH, Nielsen S, Agre P, Ottersen OP. (1998) “Aquaporin-4 water channel protein in the rat retina and optic nerve: polarized expression in Muller cells and fibrous astrocytes.” *J. Neuroscience*, 18:2506–2519.

Neely JD, Christensen BM, Nielsen S, Agre P. (1999) “Heterotetrameric composition of aquaporin-4 water channels.” *Biochemistry* 38(34):11156-63.
